# Use of clonidine in the management of opiate withdrawal in community patients

**DOI:** 10.1192/bjo.2021.537

**Published:** 2021-06-18

**Authors:** David Kelsey, Pierre Hoezoo, Pardeep Grewal

**Affiliations:** 1 North London Forensic Service; 2 Enable - Enfield alcohol and drug service; 3 The Grove Drug Treatment Service

## Abstract

**Aims:**

Clonidine has been used to alleviate symptoms of opiate withdrawal. No validated prescribing schedules exist for the use of Clonidine in opiate detoxification in community patients. We have devised a Clonidine prescribing schedule for adult outpatients seeking opiate detoxification.

**Background:**

Opiate cessation following prolonged use produces a central noradrenergic (NA) response in the locus coeruleus (LC), causing symptoms that can result in reinstatement of use. Pharmacotherapies for withdrawal are thought to work through decreased NA release in the LC by agonising pre-synaptic alpha-2 adrenoceptors. Clonidine has been used since the 1970s. However, it is off-license in the UK, and superseded by Lofexidine. Though both cause hypotension, this is less marked with Lofexidine, which may be anxiolytic and considered better tolerated. Lofexidine is no longer available in the UK. Specialists may need to resort to Clonidine for those seeking opiate detoxification.

**Method:**

We performed a feasibility study with the primary outcome being tolerability of an outpatient clonidine schedule. Patients (n = 7) were aged between 18 and 65 years (mean 32). Six were prescribed buprenorphine as opiate substitution (OST), and one methadone.

Exclusion criteria were in keeping with BNF contraindications.

An ECG was obtained for each patient before treatment. A urine drug screen and Clinical Opiate Withdrawal Scale were taken to confirm opiate dependence and withdrawal. Patients self-monitored withdrawal using the Subjective Opiate Withdrawal Scale and daily blood pressure measurements. Standard adjuvants for withdrawal were prescribed.

A test dose of 100mcg Clonidine was given to assess for hypotension. If tolerant they received 100mcg QDS, reducing over eight days.

Patients were contacted by their recovery worker twice during the period.

**Result:**

Five of the seven completed the course, two dropped out due to hypotension. No other adverse effects warranting discontinuation were encountered. Patients reported fatigue and light-headedness as their most troublesome side-effects. Of 3 patients who returned SOWS scores, 2 reported decline by 21/64 and 14/64 respectively. One reported an increase of 49/64 over 8 days. 3 of the 5 subjects who completed the course were not abstinent at completion, citing opiate withdrawal symptoms as causative.

**Conclusion:**

There is scope for the safe use of clonidine in the community for motivated individuals. Adequate monitoring of heart rate and blood pressure is required. Starting doses at 100mcg QDS appear well tolerated. Prescribers may wish to reduce this over a longer period to encourage completion and improve tolerability. Further research is needed.

